# Effects of antithrombotic drugs on the results of fecal immunochemical test in colorectal neoplasms screening

**DOI:** 10.1038/s41598-021-83007-4

**Published:** 2021-02-23

**Authors:** Takashi Ibuka, Seiji Adachi, Yohei Horibe, Tomohiko Ohno, Masatoshi Mabuchi, Yusuke Suzuki, Osamu Yamauchi, Eri Takada, Midori Iwama, Koshiro Saito, Masamichi Arao, Koji Takai, Hiroshi Araki, Masahito Shimizu

**Affiliations:** 1 Division for Regional Cancer Control, Gifu University Graduate School of Medicine, Gifu, Japan; 2 Department of Gastroenterology, Gifu University Graduate School of Medicine, Gifu, Japan; 3 Department of Gastroenterology/Internal Medicine, Gihoku Kosei Hospital, 1187-3 Takatomi, Yamagata, 501-2105 Japan

**Keywords:** Cancer, Gastroenterology

## Abstract

Fecal immunochemical test (FIT) is widely used as a colorectal cancer screening tool. Antithrombotic drugs may affect the screening performance of FIT for colorectal tumors. The aim of this study was to clarify the effect of antithrombotic agents on FIT accuracy in screening for colorectal neoplasms. This retrospective study enrolled a total of 758 patients who underwent both FIT and total colonoscopy. The effect of antithrombotic drugs on FIT accuracy in detecting colorectal neoplasms (CN), including colorectal cancer (CRC), advanced adenoma (AA), and non-advanced adenoma (NAA), was examined. Of the 758 patients, 144 (19%) received antithrombotic drugs (administration group). In administration group, 61/144 (42%) cases had CN [CRC:14, AA:15, NAA:32] and 217/614 (35%) cases had CN (CRC:43, AA:56, NAA:118) in non-administration group. The prevalence of CN was not significantly different between the two groups (p = 0.1157). There was no significant difference in sensitivity or specificity of the detection of all types of CN with or without taking antithrombotic drugs. Neither the positive predictive value nor negative predictive value of FIT was affected by antithrombotic drug administration. Taking antithrombotic drugs may not have a large impact on sensitivity, specificity, positive predictive value, or negative predictive value of FIT in screening for CN.

## Introduction

Colorectal cancer (CRC) is one of the most serious health care problems owing to its high incidence, morbidity, and mortality worldwide^1^. Most CRCs develop from a precancerous lesion, colorectal adenoma^2^. Therefore, early removal of these precancerous lesions is very significant to decrease CRC incidence and mortality^3,4^. In addition, highly accurate CRC screening, which can detect the early stages of colorectal neoplasms (CN), is useful to achieve this purpose^5^.

Fecal immunochemical test (FIT) for occult blood (hemoglobin) is one of the most frequently used CRC screening tools^5,6^. FIT is a noninvasive examination showing high sensitivity for advanced CN compared to guaiac fecal occult blood testing^7^. After FIT result is positive, colonoscopy is recommended for follow-up^8^. The result of quantitative FIT is also helpful when screening the high risk patients who require colonoscopy because increased concentration of fecal hemoglobin is an independent predictor for incident colorectal neoplasia^9^. Therefore, accurately reflecting the presence of CN in the results of FIT is extremely important in determining the need for colonoscopy and treatment, such as polypectomy, of these lesions.

Regular use of antithrombotic drugs, which are widely prescribed for prophylaxis of cardiovascular and cerebrovascular diseases, is associated with gastrointestinal bleeding^10^. Intake of antithrombotic drugs thus may facilitate bleeding and affect the efficacy of FIT. A recent cross-sectional study has revealed that regular use of aspirin and direct-acting oral anticoagulant (DOAC) is involved in lower positive predictive value (PPV) of FIT for detection of CRC and advanced adenoma (AA)^11^. On the other hand, it has also been reported that the usage of DOAC and aspirin did not affect the PPV of FIT for the evaluation of CN^12–14^.

In addition, the effect of antithrombotic drug administration on the sensitivity of FIT for detecting CN is drawing great attention. For instance, it is reported that usage of low-dose aspirin (LDA) may enhance the sensitivity of FIT for detecting AA^15^, whereas administration of oral aspirin prior to FIT did not significantly increase the test sensitivity for detecting advanced CN in a randomized clinical trial^16^.

When considering the results of the above clinical trials^11–14^, some aspects remain unclear about the effects of antithrombotic agents on colorectal tumor screening using FIT. Several clinical studies have also reported the PPV for colorectal tumors screening using FIT in patients taking antithrombotic drugs, but their range is wide^13,14,17,18^, while most recent meta-analysis revealed that FIT accuracy is not affected by antithrombotic agents^19^. Therefore, we considered it is important to reexamine the effects of antithrombotic drugs on the sensitivity and PPV of FIT for detecting CN in medical examination. The aim of the present study was to clarify whether antithrombotic drugs affect detecting performance of FIT for CN.

## Methods

### Patients

This study protocol was approved by the Institutional Review Board of the Gifu University Hospital and Gihoku Kosei Hospital, Gifu, Japan (27-269). Informed consent was obtained from all patients before total colonoscopy examination for clinical research. The study was carried out in accordance with the Declaration of Helsinki.

Between April 2012 and December 2016, 758 subjects at Gifu University Hospital (98 patients) and Gihoku Kosei Hospital (660 patients) were enrolled. The patients with positive FIT or symptoms such as abdominal discomfort, hematochezia, or constipation were performed colonoscopy. In addition, all patients were undergone FIT before colonoscopy.

### Data collection

Patients’ clinical characteristics (age, sex, complication with diabetes mellitus, hypertension, and hyperlipidemia) and information about antithrombotic drugs intake were obtained from electronic medical charts. Antiplatelet agents, LDA, and anticoagulants were defined as antithrombotic drugs. CN were classified into CRC, AA, and non-advanced adenoma (NAA). The definition of AA was as follows: ≥ 10 mm in size, or with a villous component, or a high-grade dysplasia.

As for FIT, patients were instructed to keep the sample collection tube (Alfresa Pharma Co., Ltd, Osaka, Japan) at room temperature according to the manufacturer’s instruction, and after delivering to the lab on the same day of colonoscopy, the samples were analyzed using a discrete automated clinical chemistry analyzer, NS-Plus C15 (Otsuka Electronics Co., Ltd, Osaka, Japan). The test was considered positive at ≥ 20 µg/g, and for 2-sample FIT if one sample tested positive, was considered positive. In addition, we used the same analyzer during the study period (2012–2016). We also completed the FITTER check list recommended by World endoscopy organization.

### Outcome measures and statistical analysis

First, the enrolled patients were divided into the administration and non-administration groups of antithrombotic drugs. Categorical variables, which were expressed as the median ± range, were compared between the two groups using a t-test. Next, the sensitivity, specificity, PPV, and negative predictive value (NPV) of FIT of the two groups in the detection of CN were calculated. Differences of these values between the groups were compared using a chi-squared. Differences were considered statistically significant at P-values < 0.05. All statistical analyses were carried out on JMP 10 (SAS Institute Inc., Cary, NC, USA).

### Statement of ethics

This study protocol was approved by the Institutional Review Board of the Gifu University Hospital and Gihoku Kosei Hospital, Gifu, Japan (27-269). The study was carried out in accordance with the Declaration of Helsinki.

## Results

### Comparison of baseline characteristics in patients taking and not taking antithrombotic drugs

**Figure 1:**
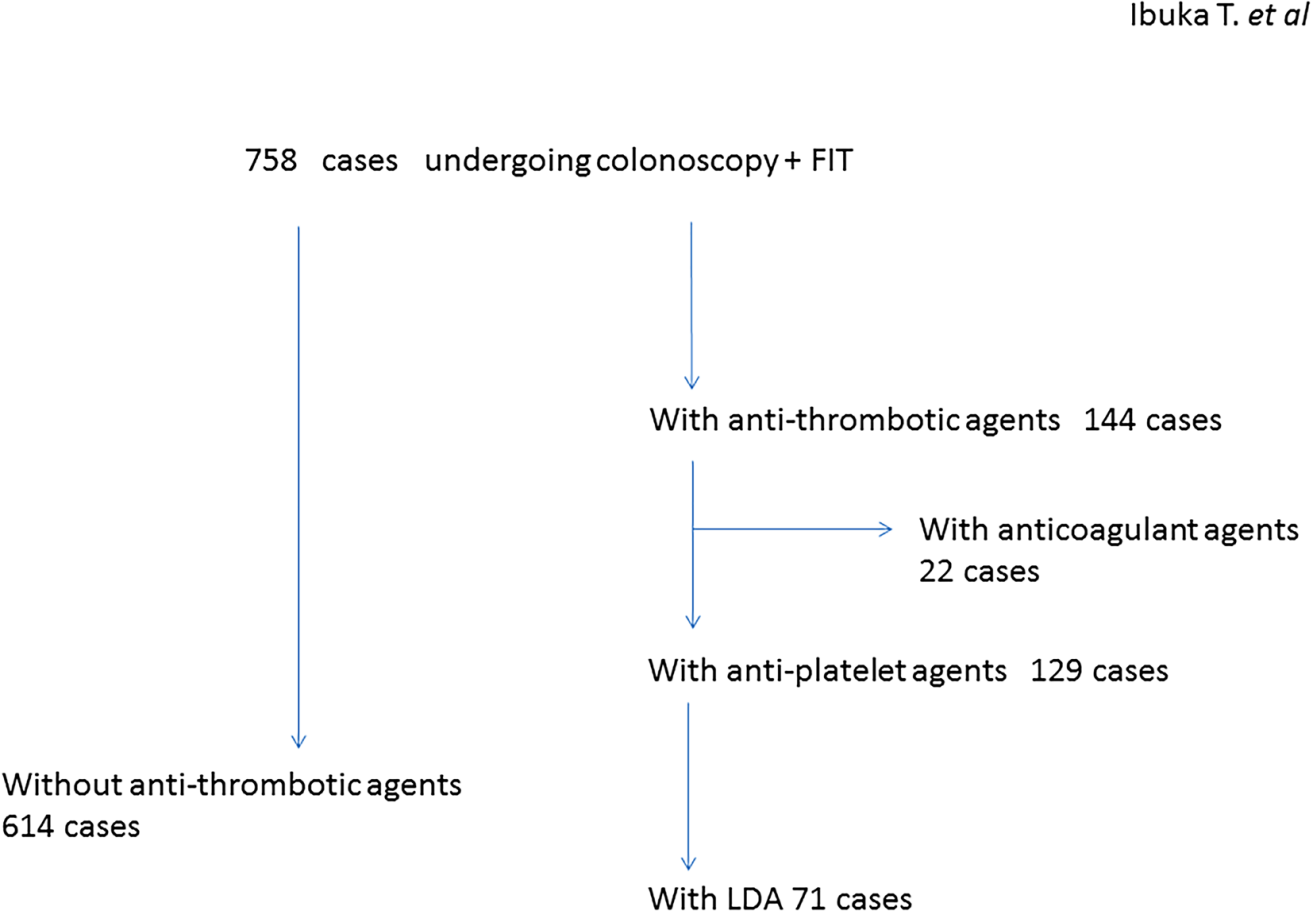
Patients flowchart. Seven cases were treated with both anti-platelet and anti-thrombotic drugs. *FIT* fecal immunochemical test, *LDA* low dose aspirin.

Among the 758 patients who underwent both FIT and total colonoscopy, 144 patients were treated with antithrombotic drugs (administration group). Among them, 129 patients received anti-platelet drugs and 71 patients of them took LDA (Fig. 1). Anticoagulant drugs (warfarin and DOAC) were given to 22 patients (with overlaps) (Table 1). As shown in Table 1, the administration group was significantly older than the non-administration group (75 years vs. 63 years, p < 0.0001) and it had more males (76.4% vs. 57.5%, p < 0.0001). In administration group, 61 cases of CN were there, including 14 of CRC, 15 of AA, and 32 of NAA. 217 cases of CN (43 of CRC, 56 of AA, 118 of NAA) were there in non-administration group. There was no significant difference between the incidences of CN development between the two groups (42.4% vs. 35.3%). Complication rates of diabetes mellitus (31.3% vs. 10.1%, p < 0.0001), hypertension (62.5% vs. 33.7%, p < 0.0001), and hyperlipidemia (31.3% vs. 17.8%, p = 0.0003) were significantly higher in the administration group compared to the non-administration group. The details of the prescription drugs were also listed in Table 1.

**Table 1:** Clinical characteristics of the 758 patients who underwent both fecal immunochemical test and total colonoscopy.

Characteristic	Anti-thrombotic agents		p-value
	Yes (n = 144)	No (n = 614)	
Age (years); median (range)	75 (46–92)	63 (21–93)	< 0.0001
Gender-Male (% of Male)	110 (76.4%)	353 (57.5%)	< 0.0001
FIT positive/negative (positive ratio)	71/73 (49.3%)	296/318 (48.2%)	0.7432
**TCS finding**			
Colorectal neoplasms	61 (42.4%)	217 (35.3%)	0.1157
Colorectal cancer	14 (9.7%)	43 (7.0%)	0.2655
Advanced adenoma	15 (10.4%)	56 (9.1%)	0.6309
Non-advanced adenoma	32 (22.2%)	118 (19.2%)	0.4155
**Complication of life-style related diseases (%)**			
Diabetes mellitus	45 (31.3%)	62 (10.1%)	< 0.0001
Hypertension	90 (62.5%)	206 (33.7%)	< 0.0001
Hyperlipidemia	45 (31.3%)	109 (17.8%)	0.0003
**Current medications (%)**			
Antiplatelet	129 (89.6%)		
Aspirin	71 (49.3%)		
Thienopyridines	29 (20.1%)		
Limaprost	21(14.6%)		
Phosphodiesterase-3 inhibitors	16 (11.1%)		
Eicosapentaenoic acid	17 (11.8%)		
Dipyridamole	6 (4.2%)		
Beraprost sodium	1 (0.7%)		
Dilazep hydrochloride hydrate	2 (1.4%)		
5-HT2	1 (0.7%)		
Anticoagulant	22 (15.3%)		
Warfarin potassium	14 (9.7%)		
DOAC	8 (5.6%)		

### Effects of antithrombotic drugs on the diagnostic performance of FIT for CN

We examined whether antithrombotic drugs affect the diagnostic performance of FIT for CN. As shown in Table 2, there was no significant difference in sensitivity or specificity of CN, CRC, CRC plus AA, and NAA, with or without oral medication of antithrombotic drugs. However, the sensitivity tended to be higher regarding CN (62.3% vs. 56.0%, p = 0.3964) and NAA (53.1% vs. 39.8%, p = 0.1775) in the administration group compared to the non-administration group. There was also no significant difference in PPV or NPV; however, the PPV tended to be higher in regard to CN (53.5% vs. 41.2%, p = 0.0604) and NAA (34.0% vs. 21.3%, p = 0.0556) in the administration group compared to the non-administration group.

**Table 2:** Effects of antithrombotic drugs on the results of fecal immunochemical test in colorectal neoplasms screening.

Measure	Outcome	Anti-thrombotic agents		p-value
		Yes (n = 144)	No (n = 614)	
Sensitivity	CN	38/61 (62.3%) CI (49.0–74.4%)	122/217 (56.0%) CI (49.3–62.9%)	0.3964
	CRC	11/14 (78.6%) CI (49.2–95.3%)	35/43 (81.4%) CI (66.6–91.6%)	0.8161
	CRC + AA	21/29 (72.4%) CI (52.8–87.3%)	75/99 (75.8%) CI (66.1–83.8%)	0.7146
	NAA only	17/32 (53.1%) CI (34.7–70.9%)	47/118 (39.8%) CI (30.9–49.3%)	0.1775
Specificity	CN	50/83 (60.2%) CI (48.9–70.8%)	223/397 (56.2%) CI (51.1–61.1%)	0.4960
	CRC	70/130 (53.9%) CI (44.9–62.6%)	310/571 (54.3%) CI (50.1–58.4%)	0.9268
	CRC + AA	65/115 (56.5%) CI (47.0–65.7%)	294/515 (57.1%) CI (52.7–61.4%)	0.9118
	NAA only	50/83 (60.2%) CI (48.9–70.8%)	223/397 (56.2%) CI (51.1–61.1%)	0.4960
PPV	CN	38/71 (53.5%) CI (41.3–65.5%)	122/296 (41.2%) CI (35.6–47.1%)	0.0604
	CRC	11/71 (15.5%) CI (8.0–26.0%)	35/296 (11.8%) CI (8.4–16.1%)	0.4018
	CRC + AA	21/71 (29.6%) CI (19.3–41.6%)	75/296 (25.3%) CI (20.5–30.7%)	0.4654
	NAA only	17/50 (34.0%) CI (21.2–48.8%)	47/221 (21.3%) CI (16.1–27.3%)	0.0556
NPV	CN	50/73 (68.5%) CI (56.6–78.9%)	223/318 (70.1%) CI (64.8–75.1%)	0.7840
	CRC	70/73 (95.9%) CI (88.5–99.1%)	310/318 (97.5%) CI (95.1–98.9%)	0.4576
	CRC + AA	65/73 (89.0%) CI (79.5–95.1%)	294/318 (92.5%) CI (89.0–95.1%)	0.3376
	NAA only	50/65 (76.9%) CI (64.8–86.5%)	223/294 (75.9%) CI (70.5–80.6%)	0.8545

*CN* colorectal neoplasms, *CRC* colorectal cancer, *AA* advanced adenoma, *NAA* non advanced adenoma, *PPV* positive predictive value, *NPV* negative predictive value, *CI* confidence interval.

We next performed a similar analysis for antiplatelet drugs (Table 3) and LDA (Table 4) administered groups. Neither anti-platelet drugs nor LDA administrations affected the sensitivity, specificity, PPV, and NPV of FIT for the detection of CN. However, in patients treated with antiplatelet drugs, the PPV showed a high tendency regarding CN (51.7% vs. 41.2%, p = 0.1360) and NAA (32.6% vs. 21.3%, p = 0.1080) in the administration group compared to the non-administration group. The data of anticoagulant drugs was not examined separately since the number of registered patients was small in the study (n = 22).

**Table 3:** Effects of antiplatelet drugs on the results of fecal immunochemical test in colorectal neoplasms screening.

Measure	Outcome	Anti-platelet agents		p-value
		Yes (n = 129)	No (n = 614)	
Sensitivity	CN	31/53 (58.5%) CI (44.1–71.9%)	122/217 (56.0%) CI (49.3–62.9%)	0.7650
	CRC	8/10 (80.0%) CI (37.7–56.5%)	35/43 (81.4%) CI (66.6–91.6%)	0.9191
	CRC + AA	17/24 (70.8%) CI (48.9–87.4%)	75/99 (75.8%) CI (66.1–83.8%)	0.6182
	NAA only	14/30 (46.7%) CI (28.3–65.7%)	47/118 (39.8%) CI (30.9–49.3%)	0.4970
Specificity	CN	47/76 (61.8%) CI (50.0–72.8%)	223/397 (56.2%) CI (51.1–61.1%)	0.3602
	CRC	67/119 (56.3%) CI (46.9–65.4%)	310/571 (54.3%) CI (50.1–58.4%)	0.6884
	CRC + AA	62/105 (59.0%) CI (52.7–61.4%)	294/515 (57.1%) CI (52.7–61.4%)	0.6563
	NAA only	47/76 (61.8%) CI (50.0–72.8%)	223/397 (56.2%) CI (51.1–61.1%)	0.3602
PPV	CN	31/60 (51.7%) CI (38.4–64.8%)	122/296 (41.2%) CI (35.6–47.1%)	0.1360
	CRC	8/60 (13.3%) CI (5.9–24.6%)	35/296 (11.8%) CI (8.4–16.1%)	0.7436
	CRC + AA	17/60 (28.3%) CI (17.5–41.4%)	75/296 (25.3%) CI (20.5–30.7%)	0.6289
	NAA only	14/43 (32.6%) CI (19.1–48.5%)	47/221 (21.3%) CI (16.1–27.3%)	0.1080
NPV	CN	47/69 (68.1%) CI (55.8–78.8%)	223/318 (70.1%) CI (64.8–75.1%)	0.7418
	CRC	67/69 (97.1%) CI (89.9–99.6%)	310/318 (97.5%) CI (95.1–98.9%)	0.8558
	CRC + AA	62/69 (89.9%) CI (80.2–95.8%)	294/318 (92.5%) CI (89.0–95.1%)	0.4712
	NAA only	47/63 (74.6%) CI (62.1–84.7%)	223/294 (75.9%) CI (70.5–80.6%)	0.8343

*CN* colorectal neoplasms, *CRC* colorectal cancer, *AA* advanced adenoma, *NAA* non advanced adenoma, *PPV* positive predictive value, *NPV* negative predictive value, *CI* confidence interval.

**Table 4:** Effects of low dose aspirin (LDA) on the results of fecal immunochemical test in colorectal neoplasms screening.

Measure	Outcome	LDA		p-value
		Yes (n = 71)	No (n = 614)	
Sensitivity	CN	14/25 (56.0%) CI (34.9–75.6%)	122/217 (56.0%) CI (49.3–62.9%)	0.9832
	CRC	5/6 (83.3%) CI (35.9–99.6%)	35/43 (81.4%) CI (66.6–91.6%)	0.9086
	CRC + AA	8/13 (61.5%) CI (35.9–99.6%)	75/99 (75.8%) CI (66.1–83.8%)	0.2370
	NAA only	6/12 (50.0%) CI (21.1–99.6%)	47/118 (39.8%) CI (30.9–49.3%)	0.4946
Specificity	CN	26/46 (56.5%) CI (41.1–71.1%)	223/397 (56.2%) CI (51.1–61.1%)	0.9638
	CRC	36/65 (55.4%) CI (42.5–67.7%)	310/571 (54.3%) CI (50.1–58.4%)	0.8668
	CRC + AA	32/58 (55.2%) CI (41.5–68.3%)	294/515 (57.1%) CI (52.7–61.4%)	0.7801
	NAA only	26/46 (56.5%) CI (41.1–71.1%)	223/397 (56.2%) CI (51.1–61.1%)	0.9638
PPV	CN	14/34 (41.2%) CI (24.6–59.3%)	122/296 (41.2%) CI (35.6–47.1%)	0.9964
	CRC	5/34 (14.7%) CI (5.0–31.1%)	35/296 (11.8%) CI (8.4–16.1%)	0.6259
	CRC + AA	8/34 (23.5%) CI (10.7–41.2%)	75/296 (25.3%) CI (20.5–30.7%)	0.8179
	NAA only	6/26 (23.1%) CI (9.0–43.6%)	47/221 (21.3%) CI (16.1–27.3%)	0.8316
NPV	CN	26/37 (70.3%) CI (53.0–84.1%)	223/318 (70.1%) CI (64.8–75.1%)	0.9854
	CRC	36/37 (97.3%) CI (85.8–99.9%)	310/318 (97.5%) CI (95.1–98.9%)	0.9454
	CRC + AA	32/37 (86.5%) CI (71.2–95.5%)	294/318 (92.5%) CI (89.0–95.1%)	0.2098
	NAA only	26/32 (81.2%) CI (63.6–92.8%)	223/294 (75.9%) CI (70.5–80.6%)	0.4946

*CN* colorectal neoplasms, *CRC* colorectal cancer, *AA* advanced adenoma, *NAA* non advanced adenoma, *PPV* positive predictive value, *NPV* negative predictive value, *CI* confidence interval.

## Discussion

FIT is accepted as a useful screening tool in reducing the mortality rate of CRC^5,6^. Currently antithrombotic drugs are widely used for preventing thrombosis in cardiovascular and cerebrovascular diseases. In the present study, the patients receiving antithrombotic drugs were significantly older and had diabetes mellitus, hypertension, and hyperlipidemia. It is well known that lifestyle-related diseases such as diabetes mellitus are significant risk factors for developing CRC^20^. Therefore, the patients who are taking antithrombotic drugs due to lifestyle-related diseases are more likely to develop colorectal tumors and require more careful screening.

Theoretically, administration of antithrombotic drugs could have the opposite effect in CN screening by FIT. The drugs may facilitate bleeding from non-advanced colorectal neoplastic lesions and thus decreasing the PPV of FIT for CRC and AA. On the other hand, if the drugs stimulate bleeding from advanced colorectal neoplastic lesions, PPV may increase significantly. PPV is one of the useful indexes to evaluate whether antithrombotic agents cause gastrointestinal mucosal damage and influence false positives of FIT.

In the present study, administration of antithrombotic drugs did not affect the sensitivity, specificity, PPV, and NPV of FIT for the detection of CN. These findings were consistent with those of previous reports showing that PPV for CN was not affected by the intake of antithrombotic drugs^12–14^. The PPVs for all colorectal neoplasia (53.5%), CRC (15.5%), CRC plus AA (29.6%), and NAA (34.0%) in patients taking antithrombotic drugs were almost the same levels as those of LDA in the previous reports (17.3–57.0%)^13,14,17,18^. Therefore, FIT is unlikely to be affected by antithrombotic drugs such as LDA, indicating that there is little need for cessation of these drugs before the test. On the other hand, a recent large cohort study has reported that both aspirin and DOAC users showed lower PPV for CRC and AA compared to non-users of these drugs^11^. In the study, the PPV for CRC (0.9% vs. 6.8%) and that of AA (20.5% vs. 32.4%) in DOAC users were especially lower compared to the matched non-users^11^. Therefore, the impact of antithrombotic drugs on FIT, especially in DOAC, needs further examination. Moreover, although we focused on the anti-platelet drugs in the present study, it might be of interest to investigate the effects of other medications on the accuracy of FIT because a number of patients are taking other medications, such as prescriptions for diabetes mellitus, hypertension, and hyperlipidemia.

Detecting adenoma, especially in AA, is involved in the effectiveness of FIT-based CRC screening^21^. FIT can detect CRC with high sensitivity and specificity; however, these values, especially in sensitivity, are relatively decreased in the detection of AA^8,22^. Therefore, improvement of the sensitivity of AA might be able to improve the performance of FIT for CRC screening. Usage of antithrombotic drugs, which could facilitate bleeding from adenoma, is expected to enhance the sensitivity of FIT for detecting AA^15^. In the present study, the sensitivity and PPV tended to be higher in regard to all colorectal neoplasia and NAA in antithrombotic drugs administration group, although there was no statistically significant difference. Removal of as many adenomatous polyps as possible during colonoscopy leads to the prevention of CRC death^23^. This might indicate that increasing the sensitivity of NAA detection could be used to add value to CRC screening. Further studies are needed to determine whether antithrombotic agents may actually increase the sensitivity of NAA.

The present study has some limitations. First, this was a retrospective study with a small sample size. In particular, due to the limited sample size of the patients that received DOAC and anticoagulant such as warfarin, we could not do a statistical analysis on them. Further multicenter, large-scale studies should be conducted to confirm the results of the present study. Second, it is necessary to verify whether the cutoff value and the kit used in the present study were appropriate. Because the cut-off value may influence the sensitivity and PPV of CRC screening, it is necessary to set a most appropriate value. Patients were enrolled from university and general hospitals in the present study, but the prevalence of colorectal tumors may differ between these hospitals. This is also one of the limitations because the prevalence is a major factor affecting PPV.

In conclusion, the results of our study suggest that antithrombotic drugs may not have a large impact on FIT sensitivity, specificity, PPV, or NPV in screening for CN. Therefore, in patients who take antithrombotic drugs, there may not be as many false positives as previously thought.

## Abbreviations

CRC: Colorectal cancer
FIT: Fecal immunochemical test
CN: Colorectal neoplasms
DOAC: Direct-acting oral anticoagulant
PPV: Positive predictive value
AA: Advanced adenoma
LDA: Low-dose aspirin
NAA: Non-advanced adenoma
NPV: Negative predictive value
